# Prehospital Trauma Scene and Transport Times for Pediatric and Adult Patients

**DOI:** 10.5811/westjem.2019.11.44597

**Published:** 2020-02-21

**Authors:** Nicklaus P. Ashburn, Nella W. Hendley, Ryan M. Angi, Andrew B. Starnes, R. Darrell Nelson, Henderson D. McGinnis, James E. Winslow, David M. Cline, Brian C. Hiestand, Jason P. Stopyra

**Affiliations:** Wake Forest School of Medicine, Department of Emergency Medicine, Winston-Salem, North Carolina

## Abstract

**Introduction:**

Increased out-of-hospital time is associated with worse outcomes in trauma. Sparse literature exists comparing prehospital scene and transport time management intervals between adult and pediatric trauma patients. National Emergency Medical Services guidelines recommend that trauma scene time be less than 10 minutes. The objective of this study was to examine prehospital time intervals in adult and pediatric trauma patients.

**Methods:**

We performed a retrospective cohort study of blunt and penetrating trauma patients in a five-county region in North Carolina using prehospital records. We included patients who were transported emergency traffic directly from the scene by ground ambulance to a Level I or Level II trauma center between 2013–2018. We defined pediatric patients as those less than 16 years old. Urbanicity was controlled for using the Centers for Medicare and Medicaid’s Ambulance Fee Schedule. We performed descriptive statistics and linear mixed-effects regression modeling.

**Results:**

A total of 2179 records met the study criteria, of which 2077 were used in the analysis. Mean scene time was 14.2 minutes (95% confidence interval [CI], 13.9–14.5) and 35.3% (n = 733) of encounters had a scene time of 10 minutes or less. Mean transport time was 17.5 minutes (95% CI, 17.0–17.9). Linear mixed-effects regression revealed that scene times were shorter for pediatric patients (p<0.0001), males (p=0.0016), penetrating injury (p<0.0001), and patients with blunt trauma in rural settings (p=0.005), and that transport times were shorter for males (p = 0.02), non-White patients (p<0.0001), and patients in urban areas (p<0.0001).

**Conclusion:**

This study population largely missed the 10-minute scene time goal. Demographic and patient factors were associated with scene and transport times. Shorter scene times occurred with pediatric patients, males, and among those with penetrating trauma. Additionally, suffering blunt trauma while in a rural environment was associated with shorter scene time. Males, non-White patients, and patients in urban environments tended to have shorter transport times. Future studies with outcomes data are needed to identify factors that prolong out-of-hospital time and to assess the impact of out-of-hospital time on patient outcomes.

## INTRODUCTION

Trauma is the leading cause of death in the United States (US) for individuals under 45 years of age.^1^,^2^ It accounts for 60% of deaths in patients less than 20 years old.^3^,^4^ Owing to the significant burden of disease, the US maintains a robust trauma care infrastructure, including trauma centers, trauma prevention programs, and emergency medical services (EMS).^5^,^6^ EMS is tasked with providing prehospital emergency care and with transporting patients to definitive care.^7^,^8^

In caring for trauma patients, out-of-hospital time is an important factor in patient outcomes.^7^–^11^ The golden hour is well-known to EMS providers and directs them to deliver trauma patients to definitive care within 60 minutes of injury.^9^,^10^ The golden hour concept is primarily attributed to R. Adams Cowley, the physician who founded Baltimore’s Shock Trauma Institute. He wrote, albeit anecdotally at the time, that “the first hour after injury will largely determine a critically-injured person’s chances for survival.”^10^ Multiple research studies support the concept that less time to definitive care results in better patient outcomes,^6^,^12^–^14^ particularly with certain disease states, such as severe head injury,^15^,^16^ abdominal injury,^17^ and thoracic injury.^18^,^19^

Sampalis et al suggest that for each additional minute of prehospital time, the risk of dying increases by 5%.^12^ Brown et al examined 164,000 trauma registry cases. In a logistic regression model, they found that prolonged scene time was associated with increased mortality among patients with hypotension, penetrating trauma, and flail chest.^20^ In a separate study, Sampalis et al performed a case-control multivariable logistic regression analysis of 360 trauma patients. They found that out-of-hospital time in excess of 60 minutes was associated with a three-fold increase in mortality.^12^ Feero et al examined nearly 1000 trauma registry cases in Oregon and concluded that less out-of-hospital time was associated with increased survival.^13^ This body of literature supports the golden hour principle.

Owing to this, an emphasis on limiting prehospital time permeates EMS care systems.^7^,^8^ Prehospital professionals are generally expected to keep trauma scene times under 10 minutes and may transport patients using emergency lights and sirens to reduce total out-of-hospital time.^7^,^8^ While well studied in the adult population, the role of the golden hour in pediatric trauma is unclear. To date, no literature specifically compares prehospital time intervals of adult to pediatric trauma patients. The purpose of this study was to examine prehospital time patterns to better understand scene and transport time practices among patients with blunt or penetrating trauma.

## METHODS

### Study Design

We performed a retrospective, regional, multijurisdictional cohort study of blunt and penetrating trauma patients in a five-county region in North Carolina (NC). We included patients who were transported directly from the scene by ground ambulance emergency traffic to a Level I or Level II trauma center between 2013 and 2018. The Wake Forest University Institutional Review Board approved this investigation and waived the requirement for informed consent. The STrengthening the Reporting of OBservational studies in Epidemiology (STROBE) guidelines helped direct the research and publication process.^21^

**Population Health Research Capsule**

What do we already know about this issue?

Time to definitive care is an important consideration in prehospital (EMS) trauma care. EMS agencies are expected to keep scene time less than 10 minutes.

What was the research question?

To characterize EMS scene and transport time practices among trauma patients in pediatric and adult cohorts

What was the major finding of the study?

EMS largely misses the 10-minute scene time goal. Pediatrics, males, and penetrating trauma patients have shorter scene times.

How does this improve population health?

Prehospital agencies and medical directors can use these results to investigate their own performance and initiate quality improvement programs.

### Study Setting

This study was conducted across five counties with Advanced Life Support (ALS) EMS agencies over a five-year period (January 1, 2013–January 1, 2018) in a mixed urban and rural area of NC. Two of the five study counties have robust urban centers with approximately 250,000 people each, while the remaining three counties are largely suburban and rural communities. The EMS agencies serve a combined population of nearly 700,000 people and transport to two American College of Surgeons (ACS)-verified Level I trauma centers and one ACS-verified Level II trauma center. Each county operates its own single-tier, ALS-level EMS agency that receives medical direction from emergency physicians with subspecialty board certification in EMS.

Each agency uses the same prehospital electronic medical record system (ESO Solutions, Austin, TX,). An EMS agency representative in each county extracted the data. These EMS representatives were blinded to the specific aims of the study. They were provided with a standardized, pilot-tested data extraction report to be run through their EMR system. The extractors did not alter the raw data. We collated each agency’s data into a single report for analysis.

### Participant Selection

We included blunt and penetrating trauma patients of all ages who were transported directly from the scene to a Level I or Level II trauma center by ground ambulance with emergency lights and sirens. These patients were identified based off a prehospital primary or secondary impression of trauma. In order to study the highest acuity trauma patients, we included only patients who were transported emergency traffic with lights and sirens. In the study region, <10% of trauma encounters are transported emergency traffic. The decision to transport emergency traffic is based on paramedic gestalt. Prisoners and patients who were declared dead in the field were excluded. We defined pediatric patients as being less than 16 years old and adult patients as being at least 16 years old. Interfacility transports were not included.

### Outcomes

The primary outcomes of this study were scene time and transport time. Scene time was defined as the moment EMS arrived on scene to the moment transport was initiated to the trauma center. Transport time was defined as the time from scene departure to trauma center arrival. EMS providers recorded scene arrival, scene departure, and trauma center arrival times in the computer automated dispatch system by selecting the appropriate digital button in the ambulance-based mobile data terminal or by radioing dispatch command, which recorded the time.

### Variables

Variables included the following: EMS agency; patient age, gender, race, and ethnicity; encounter year; primary and secondary impression; EMS on-scene time; EMS scene departure time; urbanicity defined either as urban or rural; and trauma center arrival time. Time from arrival on scene to “patient contact” was not reliably available. If patients were Hispanic or Latino, their race was considered “other.” This resulted in a three-level variable for race/ethnicity, consisting of White, African American, and other.

We used the prehospital provider’s primary and secondary impression to determine the mechanism of injury, which was defined as being either blunt or penetrating trauma. Blunt trauma included impressions of assault, bike accident, explosive incident, fall, motor vehicle accident, non-motorized vehicle accident, railway incident, and “being struck by an object.” Penetrating trauma included impressions of gunshot wound, stabbing, cutting, and “being struck by a sharp object.”

To account for urbanicity, we used the Centers for Medicare and Medicaid Services Ambulance Fee Schedule (AFS).^21^ The AFS is a nationwide descriptor for urbanicity. Locales are described as “urban,” “rural,” or “super rural” based on zip code. Each encounter was linked to its respective AFS urbanicity descriptor. No encounters in the study region were associated with a super-rural descriptor.

### Statistical Analysis

We used descriptive statistics and mixed effects modeling to characterize the sample. EMS agency, age, gender, race/ethnicity, mechanism of injury, and urbanicity were treated as categorical variables. Categorical variables were compared using Fisher’s exact test. We treated scene time and transport time as continuous variables. If scene or transport time was missing, then we excluded the record from the analysis. Scene time outliers were defined as a scene time 1.5 times the interquartile range (IQR) above the upper quartile scene time. Transport time outliers were defined as a transport time 1.5 times the IQR above the agency-specific upper quartile transport time. Outliers were excluded from the analysis.

We performed linear mixed-effects regression modeling for scene time and transport time, controlling for age, mechanism of injury, EMS agency, gender, race/ethnicity, and urbanicity. EMS agency was treated as a group random-effect variable. The encounter year was assessed for association with scene time and transport time and was not significant in either. Encounter year was excluded from the model. We created biologically plausible interaction terms via the product method and tested in the model for significance (Appendix A). Only significant interaction terms were included and reported in the models. Otherwise, we reported the full models without stepwise reduction of terms. We considered statistical differences to be significant if the probability of a type 1 error was <5% (p<0.05). The sample size was fixed, so formal power calculations were not performed. However, given the number of observations relative to the number of degrees of freedom of the covariates in the model, the model did not risk being overfit. We used SAS University Edition (SAS Institute Inc., Cary, NC) to conduct statistical analyses.

## RESULTS

### Overview

**Figure 1:**
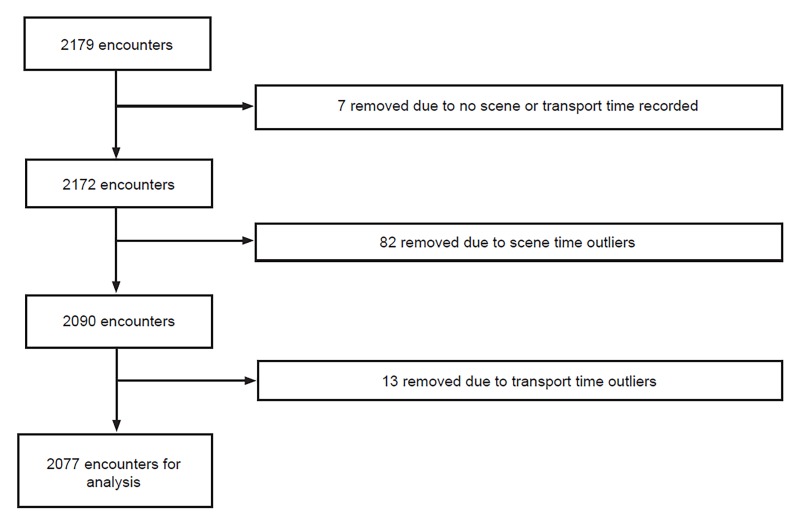
Case selection flow diagram.

A total of 2179 records met the study criteria, of which 2077 were used in the analysis (Figure 1). Of these, 92.4%% (n = 1919) were adult and 7.6% (n = 158) were pediatric. Blunt injury accounted for 80.6% (n = 1675) and penetrating injury 19.4% (n = 402). Males accounted for 68.8% (n = 1428) of the sample. White patients accounted for 62.2% (n = 1228) of the sample. Encounters occurred in rural environments 20.1% of the time (n = 416). Patient characteristics and the prevalence of the three most common mechanisms of injury are shown by age group in Table 1.

**Figure 2:**
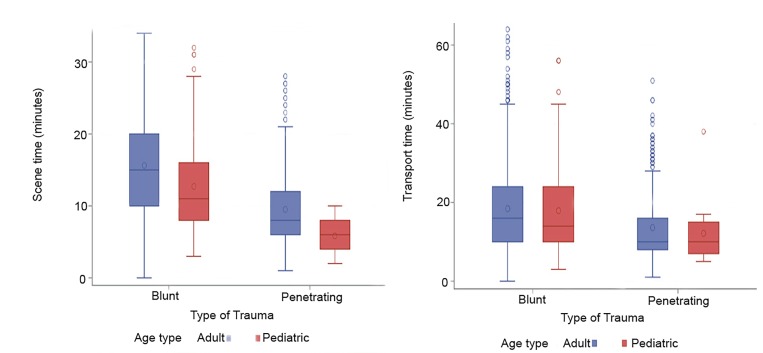
Box plots displaying scene time and transport time by mechanism of injury and age.

Overall mean scene time was 14.2 minutes (95% confidence interval [CI], 13.9–14.5). The 90^th^ percentile overall scene time was 25.0 minutes. Scene time was 10 minutes or less in 35.3% (n = 733) of encounters. Adult blunt trauma scene time (15.6 minutes, 95% CI, 15.3–16.0) was significantly greater than pediatric blunt trauma scene time (12.7 minutes, 95% CI, 11.6–13.7). Penetrating trauma scene time for adult patients (9.5 minutes, 95% CI, 9.0–10.0) was significantly greater than in pediatric patients (5.9 minutes, 95% CI, 4.6–7.2) (Figure 2).

Table 2 shows the linear mixed-effects regression model for scene time, which demonstrated that scene time was shorter for pediatric patients, penetrating injury, males, and victims of blunt trauma in rural settings. Urbanicity and race/ethnicity were not associated with scene time. The only interaction term with a significant effect on the model was the interaction of blunt mechanism with a rural location. A list of tested interaction terms is in Appendix A.

Overall mean transport time was 17.5 minutes (95% CI, 17.0–17.9). Adult blunt trauma transport time (18.4 minutes, 95% CI, 17.9–19.0) was comparable to pediatric blunt trauma transport time (17.9 minutes, 95% CI, 16.0–19.8). Adult penetrating trauma transport time (13.5 minutes, 95% CI, 12.7–14.4) was comparable to pediatric penetrating trauma transport time (12.1 minutes, 95% CI, 7.6–16.7) (Figure 2).

Table 3 shows the linear mixed-effects regression model for transport time, which demonstrated that being male or non-White and living in an urban area were associated with shorter transport times. Age and mechanism of injury were not associated with an effect on transport time. Interactions between race and urbanicity as well as gender and age were associated with a significant effect on transport time. A list of tested interaction terms is in Appendix A.

## DISCUSSION

The primary findings of this novel prehospital study are that scene times are shorter for pediatric patients, males, victims of penetrating trauma, and patients with blunt trauma in rural settings, while transport times are shorter for males, non-White patients, and patients in urban areas. Importantly, we found that the 10-minute scene time goal is achieved in only about one-third of encounters. Identifying these variances in care may enable EMS medical directors and prehospital professionals to work toward more expeditious scene and transport times for all patients, perhaps improving outcomes.

There is limited research comparing prehospital trauma scene and transport management practices in children to adults. This is the first study to show that pediatric patients have less scene time than their adult counterparts. Although our study was not designed to understand why this difference exists, there are several plausible explanations. It is possible that providers are hesitant to perform invasive field interventions in children, leading to reduced scene time. It is also likely that children may be easier to extract from difficult situations due to smaller body habitus. Penetrating trauma patients have less scene time than blunt trauma patients, perhaps owing to the perceived critical status of a gunshot or stab wound. This difference might also be explained by the likely difficulty of safely extricating a blunt trauma patient, particularly from a motor vehicle accident, whereas penetrating trauma victims are more easily loaded into the ambulance, provided that the scene is safe.

Interaction term testing revealed that blunt trauma patients in rural environments have shorter scene times. This is likely because the extended response time for rural ambulances gives rural first responders more time to extricate and prepare patients for EMS transport.^22^ Therefore, when EMS arrives on scene, the patient is closer to being ready for transport than they might otherwise be in an urban environment, thereby facilitating a shorter scene time. Regarding transport time, as expected, encounters in urban environments have shorter transport times. Similarly, non-White patients also have shorter transport times, likely due to this population’s high urban density. These shorter transport times are reasonable given the close proximity of the trauma centers to the urban population clusters. Finally, it is unclear why scene and transport times are shorter for males than females. Future study will be needed to understand these important gender differences.

To improve mortality, prehospital professionals are expected to minimize trauma scene and transport times per national and the state of North Carolina EMS guidelines.^7^,^8^ Both guidelines state that scene time should be 10 minutes or less.^7^,^8^ Within our study, the mean scene time is 14.2 (95% CI, 13.9–14.5) minutes with a 90^th^ percentile scene time of 25.0 minutes, which is nearly triple the guideline-recommended target. Only 35.3% of encounters achieve the 10-minute scene time goal. A recent analysis of over two million age-unspecified prehospital trauma encounters revealed a mean scene time of 18.1 ± 36.5 minutes for blunt trauma and 16.0 ± 45.3 minutes for penetrating trauma.^14^ These studies indicate that we are not meeting our self-identified scene time goals. By identifying factors associated with prolonged scene times, EMS agencies may be able to implement quality improvement programs to reduce scene times.

A likely driver of prolonged prehospital scene times is prehospital procedures, such as intravenous access, spinal motion restriction procedures, advanced airway management, and other Advanced Life Support (ALS) interventions. Seamon et al found that for each prehospital procedure, trauma patients were 2.6 times more likely to die before hospital discharge.^23^ These authors concluded that a “scoop and run” minimal prehospital intervention strategy is superior to a “stay and play” field intervention-heavy strategy.^23^ Furthermore, EMS transport itself compared to privately owned vehicle (POV) transport has been associated with increased trauma mortality. In a large Pennsylvania retrospective trauma registry analysis of >90,000 encounters, patients who arrived by EMS had an odds ratio of 1.9 (95% CI, 1.5–2.4) for death compared to patients who arrived by POV.^24^ The landmark Ontario Prehospital Advanced Life Support (OPALS) Major Trauma Study concluded that advanced life support ALS was not associated with improved survival compared to Basic Life Support and that patients with a Glasgow Coma Scale score of less than 9 who received ALS interventions had increased mortality.^25^ These studies provide further evidence that limited out-of-hospital time is associated with better outcomes in trauma, even if it means that prehospital professionals employ a “scoop and run” strategy and minimize prehospital interventions.

Although prehospital time intervals have previously been emphasized in light of the “golden hour” principle, some research suggests that prehospital time may not significantly contribute to patient outcomes after all.^9^,^26^,^27^ Newgard et al conducted one of the largest out-of-hospital time studies to date by examining 3656 trauma encounters from across 146 EMS agencies and 51 trauma centers. This study found no correlation between prehospital time and survival in adult patients.^26^ Similarly, Lerner et al analyzed nearly 2000 trauma records. They found that age and injury severity were associated with mortality. However, out-of-hospital time was not associated with mortality.^27^ While these studies argue against the golden hour concept, it is still possible that a cohort of patients benefits from decreased out-of-hospital time. It is especially important to remember that these studies include primarily adult patients and that pediatric-specific research is needed to clarify the role of the golden hour in pediatric trauma.

Additional studies should examine total scene time as well as scene time after making “patient contact.” In select circumstances, such as a scene requiring rescue operations, an EMS provider may be delayed in making actual “patient contact” due to the nature of the accident. Therefore, the current data may unintentionally misrepresent some scene times as prolonged when they are in fact reasonable and unavoidable by the mere nature of the situation.

Future studies should attempt to delineate why particular demographics are associated with shorter scene and transport times. In these future studies, obtaining prehospital procedure and intervention data will prove important, as these are likely significant contributors to increased scene time. With a better understanding of why particular demographics have reduced scene and transport time, quality improvement measures may be implemented with the ultimate goal of improving patient outcomes by reducing out-of-hospital time.

## LIMITATIONS

Our conclusions are limited by this being a retrospective and regional study, which opens it to bias and limits generalizability. While the time differences in this study are statistically significant, their clinical significance is unclear. As with most prehospital research, the EMS dataset is limited and unable to be linked with outcomes data. Additionally, the dataset did not include prehospital interventions. Due to these limitations, it is impossible to draw conclusions regarding out-of-hospital time and mortality, and we cannot examine the relationship between prehospital interventions and out-of-hospital time. Due to missing data and outliers, approximately 4.7% (n = 102) of the encounters were removed from the analysis, potentially opening the study to unintended selection bias. Of these, 93.1% (95/102) were outliers due to having prolonged scene or transport times. These were removed because the goal of this study was to better understand typical prehospital encounters, not substantive outliers.

It is important for readers to apply this study’s conclusions in the context of their own regional trauma care systems. In the coming years, it is likely that robust health data exchange systems between prehospital services and hospitals will be developed. When this occurs, more conclusive outcomes-linked prehospital times studies will be possible.

## CONCLUSION

This study population largely missed the 10-minute scene time goal. Scene time was shorter for pediatric patients, males, penetrating trauma, and patients with blunt trauma in rural settings. Transport time was shorter for males, non-White patients, and patients in urban areas. Future studies with outcomes data are needed to identify factors that prolong out-of-hospital time and to assess the impact of out-of-hospital time on patient outcomes. Prehospital agencies and medical directors should use this data to help investigate and improve their own agency’s scene and transport times.

## Supplementary Information



## Figures and Tables

**Table 1:** Descriptive statistics for the study population by age group with 95% confidence interval reported.

Characteristic	Adult (n = 1919)	Pediatric (n = 158)	Total (n = 2077)
Gender			
Male	69.2% (67.1–71.3) n = 1327	63.9% (56.4–71.4) n = 101	68.8% (66.8–70.8) n = 1428
Race			
White	63.4% (61.2–65.6) n = 1213	47.5% (39.7–55.3) n = 75	62.2% (60.1–64.3) n = 1288
African American	26.7% (24.7–28.7) n = 511	30.4% (23.2–37.6) n = 48	27.0% (25.1–28.9) n = 559
Other	9.9% (8.6–11.2) n = 190	22.2% (15.7–28.7) n = 35	10.9% (9.6–12.2) n = 225
Type of Trauma			
Blunt	79.8% (78.0–81.6%) n = 1532	90.5% (85.9–95.1) n = 143	80.6% (78.9–82.3) n = 1675
Urbanicity			
Rural	20.6% (18.8–22.5) n = 394	13.9% (9.0–20.3) n = 22	20.1% (18.4–21.9) n = 416
Mechanism of Injury			
MVC	55.0% (52.8–57.2) n = 1055	64.6% (57.1–72.1) n = 102	55.7% (53.6–57.8) n = 1157
Falls	16.9% (15.2–18.6) n = 325	16.5% (10.7–22.3) n = 26	16.9% (15.3–18.5) n = 351
GSW	13.9% (12.4–15.5) n = 267	7.6% (3.5–11.7) n = 12	13.4% (11.9–14.9) n = 279

*MVC*, motor vehicle collision; *GSW*, gunshot wound.

**Table 2:** Linear mixed-effects regression model for scene time.

Variable	Variance components estimate (minutes)	P-value
Fixed effects		
Base point (Intercept)	8.6	
Age type		
Adult	2.7 (1.7 to 3.7)	<.0001
Gender		
Female	0.9 (0.4 to 1.5)	0.0016
Race/Ethnicity		
African American	−0.7 (−1.4 to 0.04)	0.06
Other	0.01 (−0.9 to 0.9)	1.0
White	Reference	
Type of trauma		
Blunt	5.5 (4.7 to 6.3)	<.0001
Urbanicity		
Rural	1.8 (−0.3 to 3.9)	0.09
Blunt *rural	−2.8 (−4.8 to −0.9)	0.005
Random effects		
County		
County A	−1.5 (−3.8 to 0.8)	0.21
County B	−2.4 (−4.7 to −0.2)	0.04
County C	−0.5 (−2.8 to 1.8)	0.7
County D	0.6 (−1.6 to 2.9)	0.6
County E	3.8 (1.4 to 6.1)	0.002

**Table 3:** Linear mixed-effects regression model for transport time.

Variable	Variance components estimate (minutes)	P-value
Fixed effects		
Base point (Intercept)	22.0	
Age type		
Adult	−0.4 (−1.6 to 0.8)	0.5
Gender		
Female	2.3 (0.4 to 4.3)	0.02
Race/Ethnicity		
African American	−2.8 (−3.5 to −2.1)	<.0001
Other	−2.1 (−3.0 to −1.1)	<.0001
White	Reference	
Type of trauma		
Blunt	0.6 (−0.07 to 1.3)	0.08
Urbanicity		
Rural	5.0 (3.8 to 6.1)	<.0001
Adult female *rural	−2.7 (−4.7 to −0.7)	0.009
African American *rural	6.4 (3.7 to 9.0)	<.0001
Random effects		
County		
County A	−6.0 (−12.5 to 0.6)	0.07
County B	−9.3 (−15.9 to −2.8)	0.005
County C	2.5 (−4.1 to 9.0)	0.5
County D	3.3 (−1.8 to 11.2)	0.2
County E	8.1 (1.6 to 14.7)	0.01
