# Supplementary material for: Prehospital Trauma Scene and Transport Times for Pediatric and Adult Patients
Source: West J Emerg Med. 2020 Feb 21;21(2):455–62. doi: 10.5811/westjem.2019.11.44597 (PMC7081873; doi:10.5811/westjem.2019.11.44597)
Supplement: Supplementary file 1 [file wjem-21-455-s001.docx]

**Appendix A.** These interaction terms were assessed in the scene time and transport time linear regression models.

| Age * Sex |
| --- |
| Age * Race/Ethnicity |
| Age * Mechanism of Injury |
| Age * Urbanicity |
| Sex * Race/Ethnicity |
| Sex * Mechanism of Injury |
| Sex * Urbanicity |
| Race/Ethnicity * Mechanism of Injury |
| Race/Ethnicity * Urbanicity |
| Mechanism of Injury * Urbanicity |
